# Advances in the molecular mechanism of grapevine resistance to fungal diseases

**DOI:** 10.1186/s43897-024-00119-x

**Published:** 2025-01-02

**Authors:** Zhi Li, Ronghui Wu, Fangying Guo, Yuejin Wang, Peter Nick, Xiping Wang

**Affiliations:** 1 State Key Laboratory for Crop Stress Resistance and High-Efficiency Production, College of Horticulture, Northwest A&F University, Yangling, Shaanxi China; 2 Key Laboratory of Horticultural Plant Biology and Germplasm Innovation in Northwest China, Ministry of Agriculture, Northwest A&F University, Yangling, Shaanxi China; 3 Molecular Cell Biology, Botanical Institute, Karlsruhe Institute of Technology, Karlsruhe, Germany

**Keywords:** Immune response, Signaling molecule, Effector, Transcription factor, Regulation mechanism, Resistance, Molecular breeding

## Abstract

Grapevine is an important economic fruit tree worldwide, but grape production has been plagued by a vast number of fungal diseases, which affect tree vigor and the quality and yield of berries. To seek remedies for such issues, researchers have always been committed to conventional and biotechnological breeding. In recent years, increasing progress has been made in elucidating the molecular mechanisms of grape–pathogenic fungi interactions and resistance regulation. Here, we summarize the current knowledge on the molecular basis of grapevine resistance to fungal diseases, including fungal effector-mediated susceptibility and resistance, resistant regulatory networks in grapevine, innovative approaches of genetic transformation, and strategies to improve grape resistance. Understanding the molecular basis is important for exploring and accurately regulating grape resistance to fungal diseases.

**Supplementary Information:**

The online version contains supplementary material available at 10.1186/s43897-024-00119-x.

## Introduction

**Fig. 1:**
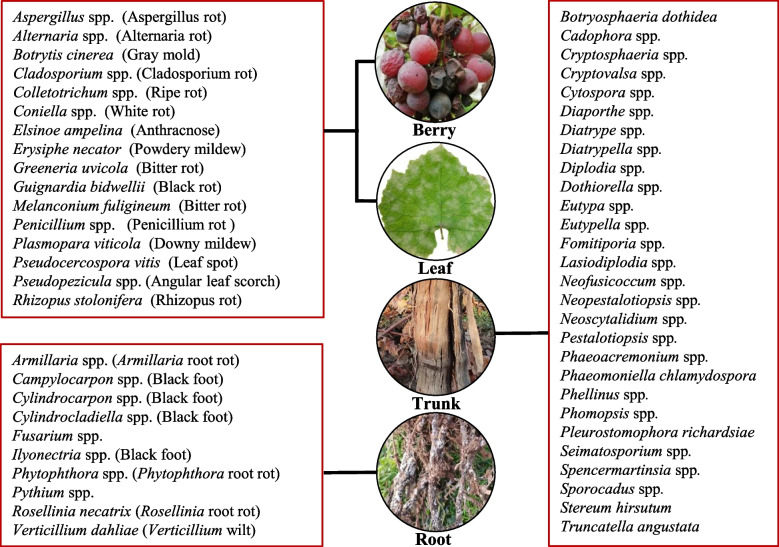
Overview of fungal and oomycete species that infect grapevine. According to the main infected tissues, grape diseases are mainly divided into three types: grapevine fruit and leaf diseases, grapevine trunk diseases (GTDs), and grapevine root diseases. The detailed fungal and oomycete species can be found in Supplemental Table S1

Grapevines are important economic fruit crops and are cultivated worldwide with a global surface area of some 7.3 million hectares, most of which are used for wine production, estimated at 232 million hectoliters (OIV 2023). However, this enormous economic potential is impacted by the high vulnerability, especially of the European Grapevine (*Vitis vinifera* L.) by fungi, bacteria, viruses, and herbivore pests such as nematodes and phylloxera (for reviews see Granett et al. 2001; König et al. 2009). Diseases caused by fungi and oomycetes account for the lion share on yield and quality loss in viticulture both, in pre- and post-harvest periods. At least 267 species distributed in 53 genera cause grape diseases in root, trunk, leaf, and fruit (Fig. 1; Table S1). The most important diseases in grapevine have been Powdery Mildew, caused by the Ascomycete *Erysiphe necator*, Downy Mildew caused by the Oomycete *Plasmopara viticola*, and Gray Mold caused by the Ascomycete *Botrytis cinerea* (for review see Armijo et al. 2016). In the aftermath of global climate change, new emerging diseases have become epidemic including Black Rot and Grapevine Trunk Diseases (GTDs) (Gramaje et al. 2018; Gutter et al. 2004; Rossi et al. 2015).

Grape fungal diseases cause significant economic losses in vineyards worldwide. Downy mildew decreases grapevine yield by about 75% in humid grapevine-producing areas, and losses by gray mold range at around 20%–50% (Fedorina et al. 2022; Koledenkova et al. 2022). In addition to production loss, fungal diseases affect the sensory properties and composition of grape juice and wine (Calonnec et al. 2004; Stummer et al. 2005).

Promoted by climate change, a new type of conditional diseases, GTDs are emerging. They are caused by at least 145 fungal species, collectively known as Esca complex diseases, Eutypa dieback, or Botryosphaeria dieback (Table S1), which lead to annual economic losses of $260 million in California, $8.3 billion in Australia, and €1 billion in France (Fontaine et al. 2016; Lorch 2014; Mondello et al. 2018). With global climate change, some main fungal diseases are forecasted to increase infection events in grapes (Bregaglio et al. 2013).

One of the main strategies to control grape fungal diseases is the application of fungicides. In Europe, around 68,000 tons of fungicides per year are used to manage grapevine diseases (Pirrello et al. 2019). Although fungicide residues are below the official maximum residue limit in different vineyards (Navarro et al. 2000; Yang et al. 2019), fungicides not only increase the resistance of pathogens but also have potential hazards to the environment, soil microorganisms, and human health (Alhanti et al. 2022; Edlinger et al. 2022; Harper et al. 2022).

An alternative strategy to reduce the application of fungicides is to develop resistant varieties of either grafts (in case of pathogens affecting foliage or bunches) or rootstocks (in case of pathogens affecting the root system). While resistance breeding has been successful in reducing fungicide load against Powdery and Downy Mildew by a factor of 5–10 fold and has become an economically relevant element of organic viticulture in Germany, France, and Switzerland, the development of these resistant varieties took several decades and initially suffered from lacking consumer acceptance (for review see Foria et al. 2022). While breeding has been strongly accelerated by marker-assisted selection (for review see Eibach et al. 2007) and will be promoted further by novel approaches, such as genomic selection (for a recent review see Magon et al. 2023), the emergence of novel pathogen strains able to breach these resistance factors (e.g., Marone Fassolo et al. 2022) shows that novel concepts of resistance management need to be integrated into breeding in order to arrive at durable solutions. However, such durable solutions will only be achieved if one considers grapevine immunity and pathogen interaction on an evolutionary scale. This review will, therefore, integrate the recent advances in grapevine pathogen interactions, and the molecular basis of defence responses into a conceptual framework of grapevine immunity. This conceptual framework is needed to interpret and valorise genetic factors to improve grapevine resistance against these diseases. Despite the vast number of grapevine pathogens which all come with their specific infection strategies, it is possible to distinguish three types of interaction that need to be briefly introduced as to understand the functional context of the molecular mechanisms contributing to resistance.

## Zigzag and beyond – conceptual framework to understand plant immunity

Plants lack the adaptive immunity found in vertebrates and rely on innate immunity. This is composed of at least two tiers that derive from co-evolution with pathogens of different lifestyles (Jones and Dangl 2006). The first tier is triggered by pattern recognition receptors (PRRs) on the cell surface that can bind to generic motifs on the pathogen surface, the pathogen-associated molecular patterns (PAMPs) through plant cell. These motifs are not only shared by entire groups of pathogens, but also are essential for their lifecycle. For instance, plant receptors against flagellin, allows to detect all flagellate bacteria, while a chitin receptor is an efficient mean to sense a fungal attack. Binding of the PAMP triggers a basal immunity, termed as PAMP-triggered immunity (PTI) culminating in the accumulation of defence compounds, so-called phytoalexins, but also enzymes attacking the pathogen, such as chitinase, or callosic plugs around the penetration site. During PTI, the attacked cell induces metabolic adjustments to contain the pathogen while sustaining the own viability.

The selective pressure on the pathogen would be expected to favor evolutionary loss of PAMPs. However, since the PAMPs are essential for the pathogen, this is not easily achieved. Instead, during prolonged co-evolution, pathogens advance by evolving effectors, proteins or small molecules that are injected into the host cell and silence basal immunity, such that the pathogen can invade and manipulate the host cell to provide resources. Upon prolonged co-evolution, some hosts developed receptors for these effectors and successfully re-installed a second tier of innate immunity. Unlike the membrane-located receptors deploying PTI, the receptors activating ETI, are intracellular, often belonging to the group of nucleotide binding domain and leucine-rich-repeat containing receptors (NLRs). The effector-triggered immunity (ETI) launched by these NLRs is usually (but not always) accompanied by a hypersensitive reaction (HR) of the attacked cell. Recent studies have shown that ETI and PTI can mutually enhance each other (Chang et al. 2022; Yuan et al. 2021) Here, the defence strategy is fundamentally different–the cell undergoes programmed cell death, sacrificing its own survival for the sake of its fellows. While PTI is an efficient strategy against necrotrophic pathogens, biotrophic pathogens are efficiently addressed by ETI. Since effectors target specific events of basal immunity, and plant receptors binding those effectors add a further level of specificity, ETI is often accompanied by a pronounced strain dependence on both sides, which is in sharp contrast to the generic nature of PTI. In this co-evolutionary context, the pathogen genes encoding those effectors mediate the detection by the host, and, thus, the avirulence of the pathogen, which is the reason, why genetic models of host-pathogen interaction have defined these effector genes as avirulence genes.

The classic two-tier concept of pathogen-host interaction (Jones and Dangl 2006) has been very useful in describing the evolutionary dynamics of plant immunity and is logically much more compelling than the old gene-for-gene concept. However, especially in the context of grapevine diseases, two elements need to be added to this model.

In addition to the classical effectors that are employed by biotrophic pathogens to suppress PTI and to reprogram the host cell to provide the intruder with nutrients, also necrotrophic pathogens can use signals to hijack host immunity. Rather than classical effectors, these signals deploy a hypersensitive cell death, which does not make any sense in the context of an attack by a necrotrophic organism. A famous example is the harpin proteins secreted by the phytopathogenic bacterium *Erwinia amylovora* that can elicit a hypersensitive cell death and are important virulence factors for this phytopathogenic, necrotrophic, bacterium (Wei et al. 1992). Likewise, *Liberibacter candidatus asiaticus*, the causative agent of the destructive Citrus Huanglongbin disease, uses chemical signals to evoke an illegitimate hypersensitive reaction. Engineering the expression of an anti-apoptotic protein from baculovirus can suppress this response and render the plant resistant (Orbović et al. 2015). Such signals are often referred to as effectors, which is not appropriate, since the term effector should be used for signals silencing PTI, and not for signals activating ETI. The alternatively used term elicitor is not wrong, but ambiguous, because it is either used for PAMPs triggering PTI, but in a biotechnological context often also as compounds triggering secondary metabolism. In the following, we will designate such compounds as programmed cell death (PCD) inducers to avoid ambiguity.

To properly address GTDs, a further group of signals needs to be considered that are not covered by the two-tier model of plant innate immunity play. The associated fungi can live in the wood for many years without causing symptoms. However, when the host plant is facing climate-change related stresses, such as heat or drought, these fungi change their behaviour and begin to secrete compounds that cause a rapid necrotic death of the host. Feeding on the dead tissue, these fungi activate a sexual cycle, form fruiting bodies and break out from the wood to propagate their spores. Thus, GTDs are conditional diseases that do not fully follow the Koch postulate (Gramaje et al. 2018), because it is not presence or absence of a pathogenic organism that decides on the development of symptoms, but it is differences in the behaviour of the pathogen what matters. The manifestation of GTDs is, thus, based on chemical communication between host and endophyte. Here, a new type of signals comes into play that is used by the pathogen to sense the dwindling health of its host and to respond by a transition from endophytic growth to a necrotrophic lifestyle. In contrast to PAMPs, effectors, or PCD inducers, this type of signals derives from the host and accumulates under stress and, therefore, act as surrender signals (Khattab et al. 2022). Similar to PAMPs, surrender signals are not generated with the *purpose* of being signals, but as byproduct of physiological processes in the sender organism itself. It is the recipient that interprets these molecules as signals. In case of PAMPs, the host cell reads them as indications of pathogen attack, in case of surrender signals, the pathogen cell reads them as indications for a severely impaired host requiring a switch in lifestyle from endophytic growth to necrotrophic sexual development. For *Neofusicoccum parvum*, a fungus associated with Botryosphaeriaceae type Dieback, the lignin precursor ferulic acid could be identified as surrender signal. Accumulation of ferulic acid conveys the information that the concurrent stilbene branch (the major phytoalexins of grapevine) is impaired. This manifestation of a weakened PTI brings the fungus to secrete Fusicoccin A, which in turn triggers programmed cell death of the plant (Khattab et al. 2022). In the absence of ferulic acid, the same fungus secretes 4-hydroxyphenylacetic acid, acting as auxin mimic stimulating growth and downmodulating a specific branch of phytoalexine synthesis (Flubacher et al. 2023). While Fusicoccin A would be a PCD inducer, 4-hydroxyphenylacetic acid would meet the criteria of an effector.

The complex signals exchanged between host and pathogen are all integrated in the context of host immunity. To understand their mode of action, it is necessary to briefly consider the actual responses underlying grapevine immunity.

## The immune responses of grapevine

Similar to other plants, PTI and ETI responses are observed during grape–pathogenic fungi interactions including Ca^2+^ influx, callose deposition, reactive oxygen species (ROS) burst, remodeling of actin, mitogen-activated protein kinases (MAPK) cascade, the activation of transcripts for phytoalexins (such as stilbene and proanthocyanidin) and pathogenesis-related proteins (PRs), and HR or PCD mediated by grape resistance (*R*) loci and fungal effectors (Brilli et al. 2018; Fu et al. 2023a; Hu et al. 2021; Luo et al. 2019; Qu et al. 2021; Wang et al. 2023d; Wingerter et al. 2021; Xu et al. 2019).

The grapevine’s immune response triggered by PAMPs is genotype-dependent. In *V*. *rupestris* cell lines, Harpin induces cell death and flg22 induces the expression of stilbene synthases (STSs), whereas in *V*. *vinifera* cell lines, Harpin does not induce death and flg22 fails to induce the expression of STSs (Chang and Nick 2012). In *V*. *rupestris* cell lines, jasmonic acid (JA) and jasmonoyl-isoleucine (JA-Ile) rapidly accumulate in response to flg22 but not to Harpin. Conversely, flg22 fails to induce JA and JA-Ile in V. vinifera cell lines (Chang et al. 2017). Subsequent cell mortality assays showed that *V*. *vinifera* is responsive to methyl jasmonate (MeJA) while* V*. *rupestris* is responsive to Harpin. The species dependent PCD induced by MeJA and Harpin in grapevine cells due to the different roles of oxidative burst (Gong et al. 2023). Additionally, both *V*. *vinifera* and *V*. *rupestris* cell lines exhibit microtubule disintegration and actin remodeling upon exposure to Harpin; however, these responses are more pronounced in *V*. *rupestris* compared with *V*. *vinifera* (Qiao et al. 2010). Cytoskeletal compounds and actin remodeling have been shown to activate grapevine defense mechanisms (Qiao et al. 2010; Sofi et al. 2023; Wang et al. 2022).

The PCD in grape plants is triggered by resistance (*R*) loci, *R* genes, regulators of grape, fungal effectors, and phytotoxins. PCD plays a crucial role in conferring resistance to powdery mildew and downy mildew. In the *Rpv3–1*, *Rpv10*, and/or *Rpv12*-genotypes, PCD occurs between 8-28 h after infection with *P*. *viticola* (Wingerter et al. 2021). Transgenic *V*. *vinifera* cultivars carrying two *R* genes *MrRUN1* and *MrRPV1* exhibit resistance against *E*. *necator* and *P*. *viticola* through the induction of PCD (Feechan et al. 2013). Grapevine metacaspase genes MC2 and MC5 are also involved in ETI-like cell death during grapevine defense against *P*. *viticola* infection, suggesting their regulatory role in PCD (Gong et al. 2019). Additionally, the effector PVITv1008311 from *P*. *viticola* triggers an immune response specifically in resistant *V*. *riparia* but not susceptible *V*. *vinifera*, indicating its recognition by the immune system of *V*. *raparia* (Brilli et al. 2018). By contrary, phytotoxin-triggered PCD exclusively benefits necrotrophic plant pathogenic fungi (Dickman and de Figueiredo 2013). For instance, fungal phytotoxin fusicoccin A is secreted from *Neofusicoccum parvum* upon activation by ferulic acid, and subsequently activates grapevine’s PCD mediated by14‐3‐3 proteins (Khattab et al. 2022). Interestingly, fungal cultures secrete 4‐hydroxyphenylacetic acid (4‐HPA) when not challenged with ferulic acid (Khattab et al. 2022). Recently, the fungal metabolite 4‐HPA from *N*. *parvum* inhibits early responses and PCD at the endophytic phase during Harpin-induced defense (Flubacher et al. 2023). These findings provide novel insights into the transition of endophytic fungi into pathogenic fungi in grapes.

## Breaching the first barrier – how fungi get in

Fungal pathogens infect host tissue through the production and secretion of cutinases, plant cell wall-degrading enzymes (PCWDEs), secondary metabolites (SMs), or effectors to obtain nutrients and complete their life cycle (Faris and Friesen 2020; Horbach et al. 2011). Phylogenomic analysis showed that the expansion of gene families is associated with plant cell wall degradation and secondary metabolism in the genomes of pathogenic fungi in grapevine (Garcia et al. 2021; Morales-Cruz et al. 2015). Similarly, a comparative genomic analysis of 40 *Eutypa lata* isolates, the causal organism of Eutypa dieback of grapevine, revealed that this species has a highly dynamic SM production potential, suggesting adaptive evolution to abiotic factors and potential host genotypes (Onetto et al. 2022). Phytotoxic metabolites (PMs), also known as SM members, are important virulence factors that assist fungi in invading and colonizing grapevines (Andolfi et al. 2011). A total of 76 PMs has been identified from fungal pathogens involved in GTDs (Reveglia et al. 2022). Although the mode of action of most PMs remains largely unknown, PMs may affect membrane integrity, inhibit the activity of plant enzymes, and interfere with normal metabolic processes (Möbius and Hertweck 2009). As a necrotrophic pathogen, *B*. *cinerea* secretes sesquiterpen botrydial and its derivatives to facilitate both penetration and colonization (Choquer et al. 2007). *B*. *cinerea* and *Sclerotinia sclerotiorum* release organic acids as virulence factors, while organic acids can acidify the host tissue, and acidic ambient pH induces the expression of polygalacturonase gene (*Bcpg3*) in *B*. *cinerea* and a number of CWDEs in *S*. *sclerotiorum* (Billon-Grand et al. 2012; Wubben et al. 2000; Xu et al. 2018). In the process of overcoming the first barrier, the effectors also play a crucial role. Pathogens degrade plant cell by secreting polygalacturonases (PGs) and plants counteract the PGs through producing PG-inhibiting proteins (PGIPs). However, *S*. *sclerotiorum* PGIP-INactivating Effector 1 (SsPINE1) interacts with and inactivates the PGIP of pea and *Arabidopsis* to enhance *S*. *sclerotiorum* virulence (Wei et al. 2022). *S*. *sclerotiorum* mainly infects grapevine shoots and causes canker-like lesions (Perelló et al. 2024). Perhaps there is a similar interference mechanism in the grape–*S*. *sclerotiorum* pathosystem.

## Breaching the second barrier – how to silence PTI

Fungal pathogens interfere with plant immunity and basal defenses via the secretion of virulence factors including small RNAs, exopolysaccharides, and effectors (Derbyshire et al. 2019; Wilson and McDowell 2022). *B*. *cinerea* small RNAs are able to suppress immunity by binding to *Arabidopsis* Argonaute 1 (AGO1) and selectively silencing host immunity genes (Weiberg et al. 2013). Additionally,* B*. *cinerea* produces an exopolysaccharide to activate SA pathway, which antagonizes JA pathway, and evade the JA-dependent resistance against necrotrophic pathogens (El Oirdi et al. 2011). In recent years, the pathogenic mechanism of effectors has been clarified compared with other pathogenic factors on grapes, especially in the interaction between grapes and *Plasmopara viticola*.

### Effector discovery of pathogenic fungi in grapevine

The identification of effectors is crucial for a thorough understanding of the pathogenic mechanism of pathogens. At present, the identification of effectors mainly depends on high-quality fungal genome sequencing (Wilson and McDowell 2022). To date, at least 32 genomes of 24 species of pathogenic fungi in grapevine have been sequenced (Table S2), including *B*. *cinerea* (Van Kan et al. 2017), *Colletotrichum viniferum* (Dou et al. 2022), *Coniella diplodiella* (Liu et al. 2021b), *Coniella vitis* (Zhou and Li 2020), *Diplodia seriata* (Robert-Siegwald et al. 2017), *Elsinoe ampelina* (Li et al. 2020b; Haridas et al. 2020), *Erysiphe necator* (Jones et al. 2014; Zaccaron et al. 2023), *E. lata* (Blanco-Ulate et al. 2013a), *Fomitiporia mediterranea* (Floudas et al. 2012), *Lasiodiplodia* spp*.* (Garcia et al. 2021; Yan et al. 2018), *Neofusicoccum* spp*.* (Blanco-Ulate et al. 2013b; Garcia et al. 2021), *Plasmopara viticola* (Brilli et al. 2018; Yin et al. 2017; Dussert et al. 2019), and *Phaeomoniella chlamydospore* (Morales-Cruz et al. 2015). Sizes of sequenced genomes vary widely, ranging from the smallest genome of *Phaeomoniella chlamydospore* ‘UCR-PC4’ of 27.5 Mb (Morales-Cruz et al. 2015) to the largest genome of *P. viticola* ‘JL-7–2’ of 101.3 Mb (Yin et al. 2017). Recently, comparative genomic analysis found abundant gene duplicates in genes encoding candidate secreted effector proteins in *E. necator* (Zaccaron et al. 2023). Although genome sequencing efforts of pathogenic fungi in grapevine have been underway since 2012, over 90% of the pathogenic fungi have not been sequenced yet.

Downy mildew of grapevine is caused by biotrophic *P*. *viticola*, one of the members of oomycetes, whose effecters usually include RxLR (Arg-x-Leu-Arg, where x is any amino acid) and CRN (crinkling and necrosis) (Fu et al. 2023a). A total of 58 and 90 CRN effectors and 151 and 57 RxLR effectors were predicted from *P*. *viticola* isolates JL-7-2 and PvitFEMO1, respectively (Brilli et al. 2018; Yin et al. 2017). Subsequently, 420 RxLR effectors were predicted from a high-quality genome assembly of *P*. *viticola* isolate INRAPV221 (Dussert et al. 2019). In addition to oomycetes, candidate effectors are often predicted from secreted proteins via classical pathways based on several features in fungi: (i) containing an N-terminal signal peptide; (ii) lacking transmembrane domains; (iii) lacking glycosyl-phosphatidyl-inositol (GPI)-anchor sites; (iv) having extracellular localization; (v) less than 300 amino acids; and (vi) containing cysteine residues (Gohari et al. 2015; Huang et al. 2022). For pathogenic fungi in grapevine, numerous effectors have been identified, such as 359 putative effectors in *L*. *theobromae* (Yan et al. 2018), 248 putative effectors in *C*. *diplodiella* (Liu et al. 2021b), and 103 putative effectors in *E. ampelina* (Li et al. 2021a, b). However, some fungal effectors are secreted via non-classical pathways instead of the membrane secretion system of the endoplasmic reticulum and the Golgi apparatus. For example, a biotrophic fungus *Ustilago maydis* may be involved in the delivery of effectors in vesicles (Ludwig et al. 2021). An effector VdSSR1 lacking a signal peptide from *Verticillium dahlia* is secreted to the plant nucleus to interfere with the nuclear export of AGO1–miRNA complexes (Zhu et al. 2022a). Therefore, other unknown effector factors may exist in grape pathogenic fungi.

### Modulating grapevine defense response by fungal effectors

**Fig. 2:**
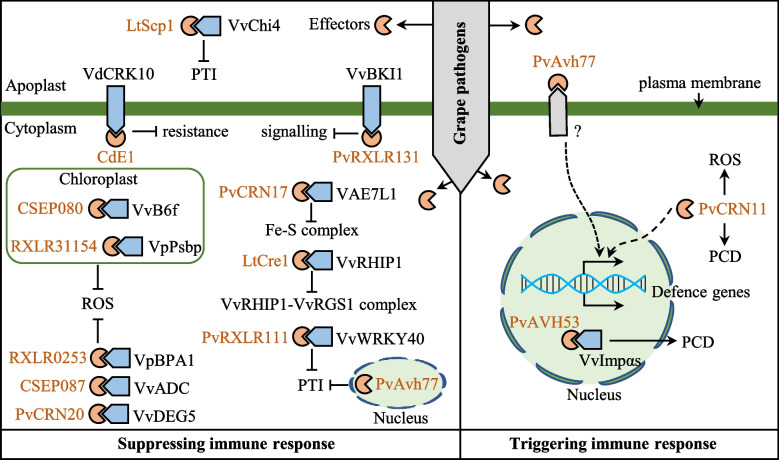
Grape targets and modes of action of fungal and oomycete effectors. Effectors are shown in orange, whereas its target proteins are in blue. The mode of action of the effectors is written in black text. Solid lines represent a verified relationship, and dotted lines represent connections that need to be characterized further. Arrows represent promotion. Ended arrows represent suppression

Effectors either promote fungal virulence by suppressing the host immune reaction or triggering HR (Lo Presti et al. 2015). At present, research on the effectors of grape pathogenic fungi mainly focuses on *P*. *viticola*, *E. necator*, *L. theobromae*, and *C*. *diplodiella* (Fig. 2; Table 1). Many fungal effectors suppress host targets that positively regulate grape immunity via disrupting PTI, such as signal transduction and ROS burst. The *P*. *viticola* effector PvRXLR131 interacts with grape BRI1 kinase inhibitor 1 (VvBKI1) to prevent brassinosteroid (BR) and ERECTA (ER) signaling and promote infection (Lan et al. 2019). Another *P*. *viticola* effector PvCRN20 disrupts grape defenses by suppressing the chloroplast import of DEG5, thereby reducing host ROS accumulation and facilitating *P*. *viticola* infection (Fu et al. 2024). LtScp1 is an effector of *L. theobromae* and its functions in the apoplast of grape cells, where it interacts with grapevine chitinase VvChi4 and interferes with the ability of VvChi4 to bind to chitin (Peng et al. 2022). The grape chloroplast localization protein VviB6f (cytochrome b6-f complex iron sulfur subunit) is targeted by *E*. *necator* effector CSEP080, leading to the reduction in ROS and pectin degradation, which enhances *E*. *necator* infection (Mu et al. 2023a). Furthermore, one of the ways in which fungal effectors exert their influence is by modulating the enzymatic activity of grape proteins. The *E*. *necator* effector CSEP087 was shown to interact with grape VviADC (arginine decarboxylase), which promotes ROS accumulation (Mu et al. 2022).

**Table 1:** List of identified and characterized effectors from pathogenic fungi and oomycetes in grapevine

**Pathogen**	**Effector name**	**Targets**	**Target name**	**Effector functions**	**Reference**
*Coniella diplodiella*	CdE1	VdCRK10	cysteine-rich receptor-like kinase 10	suppresses of VdCRK10-mediated immunity	Liu et al. 2024
*Erysiphe necator*	CSEP080	VviB6f	cytochrome b6 f complex iron sulfur subunit	affects photosynthesis	Mu et al. 2023a
		VviPE	pectinesterase	promotes pectin degradation	Mu et al. 2023a
	CSEP087	VviADC	arginine decarboxylase	inhibits ROS production	Mu et al. 2022
*Lasiodiplodia theobromae*	LtCre1	VvRHIP1	RGS1-HXK1-interacting protein 1	disrupts sugar signaling-mediated immunity	Xing et al. 2023
	LtScp1	VvChi4	chitinase	interferes VvChi4 binding to chitin	Peng et al. 2022
*Plasmopara viticola*	PvAVH53	VvImpα and VvImpα4	nuclear import factor importin alphas	promotes grape immunity	Chen et al. 2021
	PvAvh77	–	–	promotes grape immunity	Fu et al. 2023a
	PvCRN11	–	–	promotes grape immunity	Fu et al. 2023b
	PvCRN17	VvAE7	ASYMMETRIC LEAVES 1/2 ENHANCER 7 like-1	demolishes the CIA Fe-S cluster transfer complex	Xiang et al. 2022
	PvCRN20	VvDEG5	degradation of periplasmic proteins	inhibits ROS production	Fu et al. 2024
	PvRXLR111	VvWRKY40	WRKY transcription factor 40	stabilizes VvWRKY40 and promote virulence	Ma et al. 2021a
	PvRXLR131	VvBKI1	BRI1 kinase inhibitor 1	inhibits signal transduction	Lan et al. 2019
	RXLR31154	VpPsbP	oxygen-evolving enhancer 2	reduces ROS accumulation	Liu et al. 2021a
	RXLR50253	VpBPA1	binding partner of ACD11-1	inhibits ROS production and degradation of VpBPA1	Yin et al. 2022b

A critical mode of action of fungal effectors is to interfere with the formation of biologically active protein complexes to suppress grape immunity. The Crinkler effector PvCRN17 from *P*. *viticola* suppresses Fe-S protein-mediated defense responses through interaction with grape VAE7L1 (*Vitis* protein ASYMMETRIC LEAVES 1/2 ENHANCER 7-Like 1), subverting Fe-S cluster assembly and disrupting the formation of maturation of the Fe-S protein (Xiang et al. 2022). Another *L. theobromae* effector LtCre1 targets the grape VvRHIP1 (RGS1-HXK1-interacting protein 1) protein to disrupt the association of the VvRHIP1-VvRGS1 complex, interfering with sugar signaling and increasing grape susceptibility to *L*. *theobromae* (Xing et al. 2023). Fungal effectors also achieve infection via regulating the protein stability of the key components of the grape immune system. For example, a cell death-inducing effector PvRXLR111 interacts with and stabilizes the grape putative WRKY transcription factor (TF) 40 (VvWRKY40), which functions as a negative regulator in plant immunity (Ma et al. 2021a). The chloroplast localization oxygen-evolving enhancer 2 (VpPsbP) is a susceptibility factor involved in the reduction in ROS in grapevine. The *P*. *viticola* effectors RxLR31154 and RxLR50253 target and stabilize VpPsbP and VpBPA1, respectively, thereby promoting susceptibility to *P*. *viticola* in grapevine (Liu et al. 2021a; Yin et al. 2022b). By contrast, the *C*. *diplodiella* effector CdE1 plays a role in decreasing the accumulation of cysteine-rich receptor-like kinase 10 (VdCRK10) and suppressing VdCRK10-mediated immunity in *V. davidii* (Liu et al. 2024).

In addition to disrupting the host immune response, fungal effectors induce immunity depending on cellular compartments of effectors in host cells. The *P*. *viticola* effector PvAVH53 enters the nucleus and interacts with grape VvImpαs (nuclear import factor importin alphas), which are positive factors against downy mildew, inducing grape innate immunity (Chen et al. 2021). By contrast, grape immune reaction induced by the *P*. *viticola* effector PvCRN11 is not dependent on its nucleus localization but acts in a BAK1-dependent manner to enhance grape resistance to downy mildew (Fu et al. 2023b). Interestingly, the *P*. *viticola* effector PvAvh77 suppresses grape immunity in a nuclear localization-dependent manner but induces immune responses in the apoplast of susceptible ‘Thompson Seedless’ (Fu et al. 2023a). In summary, the modes of action of fungal effectors vary, and revealing their working mechanisms is the basis for accurately formulating disease resistance strategies in grapevine.

## Resistant regulatory networks in grapevine

**Fig. 3:**
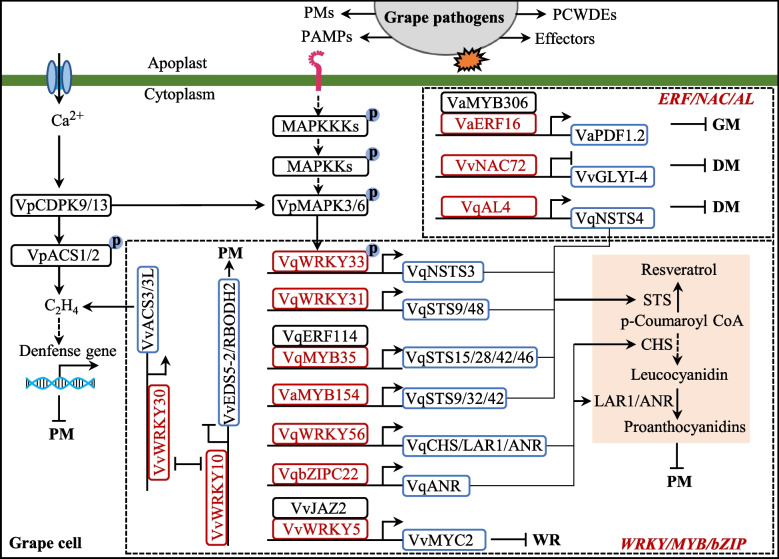
Perception and transcriptional regulation of resistance-related genes in grapevine. Fungal infection induces Ca^2+^ influx and is perceived by receptor kinases, triggering MAPK cascades and the activity of transcription factor. Transcription factors such as WRKY, MYB, NAC, and ERF contribute to disease resistance in grapevine by regulating ET-, JA-, and SA-dependent defense, as well as accumulation of phytoalexin including stilbene and proanthocyanidin. Arrows represent promotion. Ended arrows represent suppression. P in the blue circle indicates phosphorylation. DM, downy mildew; GM, gray mold; WR, white rot; PAMPs, pathogen-associated molecular patterns; PCWDEs, plant cell wall-degrading enzymes; PM, powdery mildew; PMs, phytotoxic metabolites

In recent years, accumulating evidence has revealed resistant regulatory networks by protein kinases, WRKY, MYB, ERF, bZIP, and NAC TFs, and resistance-related genes enhancing fungal disease tolerance in grapevine (Fig. 3 and S1; Table 2 and S3).

**Table 2:** Functional analysis of resistance and susceptibility-related genes in transgenic grapevine

**Gene**	**Name**	**Source**	**Genetic transformation**	**Resistance**	**Susceptibility**	**Reference**
*AtRPW8.2*	Resistance to *powdery mildew 8*	*Arabidopsis*	Overexpression	*Erysiphe necator*		Hu et al. 2021
*MrRPV1*	Resistance to *Plasmopara viticola*	*Muscadinia rotundifolia*	Overexpression	*Plasmopara viticola*		Feechan et al. 2013
*MrRUN1*	Resistance to *Erysiphe necator*	*Muscadinia rotundifolia*	Overexpression	*Erysiphe necator*		Feechan et al. 2013
*RCC2*	Chitinase	Rice	Overexpression	*Erysiphe necator* *Elisinoe ampelina*		Yamamoto et al. 2000
*VpBPA1*	Binding partner of ACD11-1	*V. piasezkii* accession ‘Liuba-8’	Overexpression		*Plasmopara viticola*	Yin et al. 2022b
*VpCDPK13*	Calcium-dependent protein kinase	*V*. *pseudoreticulata* accession ‘Baihe-35-1’	Overexpression	*Erysiphe necator*		Hu et al. 2021
*VpCDPK9*	Calcium-dependent protein kinase	*V*. *pseudoreticulata* accession ‘Baihe-35-1’	Overexpression	*Erysiphe necator*		Hu et al. 2021
*VpPR10.1*	Pathogenesis-related protein	*V*. *pseudoreticulata* accession ‘Baihe-35-1’	Overexpression	*Plasmopara viticola*		Ma et al. 2018
*VpPR4-1*	Pathogenesis-related protein	*V*. *pseudoreticulata* accession ‘Baihe-35-1’	Overexpression	*Erysiphe necator*		Dai et al. 2016
*VpPsbP*	Oxygen-evolving enhancer 2	*V. piasezkii* accession ‘Liuba-8’	Overexpression		*Plasmopara viticola*	Liu et al. 2021a
*VpPUB23*	Ubiquitin ligase	*V*. *pseudoreticulata* accession ‘Baihe-35-1’	Overexpression	*Erysiphe necator*		Zhou et al. 2014
*VpRH2*	Ubiquitin ligase	*V*. *pseudoreticulata* accession ‘Baihe-35-1’	Overexpression	*Erysiphe necator*		Wang et al. 2017
*VpSTS29/STS2*	Stilbene synthase	*V*. *pseudoreticulata* accession ‘Baihe-35-1’	Overexpression	*Erysiphe necator*		Xu et al. 2019
*VqNSTS3*	Stilbene synthase	*V*. *quinquangularis* accession ‘Danfeng-2’	Overexpression	*Erysiphe necator*		Liu et al. 2023
*VqNSTS4*	Stilbene synthase	*V*. *quinquangularis* accession ‘Danfeng-2’	Overexpression	*Erysiphe necator*		Yan et al. 2023
*VqSTS6*	Stilbene synthase	*V*. *quinquangularis* accession ‘Danfeng-2’	Overexpression	*Erysiphe necator*		Liu et al. 2019
*VvDEG5*	Degradation of periplasmic protein 5	*V*. *vinifera* cv. Pinot Noir	Overexpression	*Plasmopara viticola*		Fu et al. 2024
*VvDEG8*	Degradation of periplasmic protein 8	*V*. *vinifera* cv. Pinot Noir	Overexpression	*Plasmopara viticola*		Fu et al. 2024
*VvEDR1*	Enhanced disease resistance 1	*V. vinifera* cv. Thompson Seedless	CRISPR/Cas9	*Erysiphe necator*		Yu et al. 2024
*VviDMR6-1*	Downy Mildew Resistant 6	*V. vinifera* cv. Crimson seedless and Sugraone	CRISPR/Cas9	*Plasmopara viticola*		Giacomelli et al. 2023
*VviDMR6-2*	Downy Mildew Resistant 6	*V. vinifera* cv. Crimson seedless and Sugraone	CRISPR/Cas9	*Plasmopara viticola*		Giacomelli et al. 2023
*VvMLO3*	Mildew Locus O	*V. vinifera* cv. Thompson Seedless	CRISPR/Cas9	*Erysiphe necator*		Wan et al. 2020
*VvMLO6/7/11*	Mildew Locus O	*V. vinifera* cv. Brachetto	RNAi	*Erysiphe necator*		Pessina et al. 2016
*VvPR4b*	Pathogenesis-related protein	*V. vinifera* cv. Thompson Seedless	CRISPR/Cas9		*Plasmopara viticola*	Li et al. 2020a
*VvPUB26*	Ubiquitin ligase	*V*. *vinifera* cv. Thompson Seedless	Overexpression	*Erysiphe necator*		Zhao et al. 2024

### Protein kinases

Plant receptor protein kinases percept PAMPs or host-derived damage-associated molecular patterns (DAMPs) and trigger downstream signaling networks manipulated by MAPK cascades and calcium-activated protein kinases to control the activities of a plethora of TFs and the synthesis of enzymes, hormones, and antimicrobial chemicals (Tena et al. 2011). Oligogalacturonides (OGs), chitooligosaccharides, chitin, and chitosan act as PAMPs or DAMPs in grapevine (Brulé et al. 2019; Héloir et al. 2019). *V*. *vinifera* LysM receptor kinases (VvLYK1-1 and VvLYK1-2) participate in chitin- and chitosan-triggered immunity and restore chitooligosaccharide-induced MAPK activation and immune gene expression in *Arabidopsis* mutant *Atcerk1* (Brulé et al. 2019). Recently, VvLYK5-1 mediates the perception of chitin through its association with VvLYK1-1. VvLYK5-1 restores chitin-induced MAPK activation, defense gene expression, callose deposition against *E*. *necator* in *Arabidopsis Atlyk4/5* double mutant (Roudaire et al. 2023). VqSERK3/BAK1, a member of somatic embryogenesis receptor kinases (SERKs) from *V*. *quinquangularis*, enhances resistance to *E*. *necator* (Li et al. 2022). These results suggest that grape protein kinases play important roles in sensing pathogens and regulating disease resistance.

In grapevine, VqMAKP3 and MAPK6 are activated by *E. necator* and then phosphorylate VqWRKY33, which regulates the expression of VqSTSs and the production of stilbenes and enhances grape resistance to *E. necator* (Yan et al. 2023). Enhanced disease resistance 1 (EDR1), a Raf-like mitogen-activated protein kinase kinase kinase (MAPKKK), negatively regulates defense responses against powdery mildew in Arabidopsis (Frye and Innes 1998). Recently, grapevine VviEDR1-edited and chimeric edited lines display enhanced resistance to *E. necator* via upregulating 149 genes encoding leucine-rich repeat domain (LRR) and 56 TFs (Yu et al. 2024). Calcium ions are key second messenger ions during plant immunity, and their sensors include Ca^2+^-dependent protein kinases (CDPKs), calcineurin B-Like (CBL), and CBL-interacting protein kinase (CIPK) (Köster et al. 2022). For grape CDPKs, there are a total of 19 members in the wild Chinese grapevine *V*. *pseudoreticulata* accession Baihe-35-1 (Zhang et al. 2015). Among them, VpCDPK9 and VpCDPK13 interact with and phosphorylate VpMAPK3, VpMAPK6, VpACS1 and VpACS2, contributing to powdery mildew resistance by positively regulating SA and ethylene signaling in grapevine (Hu et al. 2021). VqbZIP1 not only activates the transcript level of STS genes but also interacts with VqSnRK2.4 and VqSnRK2.6, whose co-expression with VqbZIP1 confers higher efficiency than the expression of VqbZIP1 alone in activating the STS promoters (Wang et al. 2019a). However, whether VqSnRK2.4 and VqSnRK2.6 phosphorylate VqbZIP1 and promote its regulatory effect remain unclear. Further exploration on the resistant function of protein kinases in grape to fungal diseases is warranted.

### WRKY TFs

In plant immune response, TFs play a regulatory role for defense genes. Recent research has found that conserved TFs NRZ1 and NRM1 also regulate NLRs (Zhang et al. 2024). In particular, WRKY TFs are prominent regulators of the plant defense transcriptome and disease resistance (Eulgem and Somssich 2007). In grapevine, there are 59 WRKY genes, of which 38 respond to grape powdery mildew (Guo et al. 2014). Several WRKYs enhance grapevine powdery mildew resistance and positively regulate the synthesis of phenylpropanoid pathway metabolites, such as resveratrol and proanthocyanidin (PA). Resveratrol is a kind of stilbene phytoalexin with broad-spectrum resistance to a range of pathogens in grapes (Khattab et al. 2021), and its synthesis is determined by the key enzyme stilbene synthase (STS) with 48 members in *V*. *vinifera* (Parage et al. 2012). Overexpression of *VqNSTS3*, *VqSTS6*, and *VpSTS29/STS2* in *V*. *vinifera* increases resistance to *E. necator* (Liu et al. 2019; 2023; Xu et al. 2019). VqWRKY31 binds to the promoters of *VvSTS9* and *VvSTS48*, activates their expression, produces additional stilbene metabolites, and enhances grapevine resistance to *E. necator* (Yin et al. 2022a). VqWRKY33 is phosphorylated by VqMAPK3/VqMAPK6 and then activates *VqNSTS3* expression and enhances grapevine resistance to *E. necator* (Liu et al. 2023). Furthermore, overexpression of *VqWRKY56* in *V. vinifera* increases the proanthocyanidin content by binding to *VvCHS3*, *VvLAR1*, and *VvANR* promoters and reduces susceptibility to *E. necator* (Wang et al. 2023d). Recently, *VviWRKY10* and *VviWRKY30*, as homologous genes of *AtWRKY18* and -*40*, were found to co-regulate powdery mildew resistance in grapevine, but the regulatory mechanism in grape is different from the functional redundancy in *Arabidopsis* (Zhou et al. 2024). VviWRKY10 inhibits the expression of *VviEDS5-2*, *VviPR1*, *VviPR5*, and *VviRBOHD2* as a negative regulator of SA-dependent defense, whereas VviWRKY30 promotes the expression of VviACS3 and VviACS3L as a positive regulator in ethylene (ET)-dependent defense to *E. necator*. Interestingly, VviWRKY10 and VviWRKY30 bind each other’s promoters and inhibit each other’s expression (Zhou et al. 2024).

In addition to regulating grape powdery mildew resistance, WRKY TFs play a vital role in the regulation of grape resistance to other fungal diseases. Overexpression of *VvWRKY1* can enhance grapevine resistance to downy mildew by activating the jasmonic acid (JA) signaling pathway (Marchive et al. 2013). VvWRKY5 regulates the JA-dependent defense associated with grape white rot resistance by binding *VvJAZ2* and *VvMYC2* promoters, thereby inhibiting and activating the transcription of *VvJAZ2* and *VvMYC2*, respectively (Zhang et al. 2023b). By contrast, VvWRKY40 and VvWRKY52 negatively regulate grapevine resistance to downy mildew and gray mold, respectively (Ma et al. 2021a; Wang et al. 2018).

### Other TFs

In addition to WRKYs, many TFs participate in grape fungal disease resistance by regulating the transcription of *STSs* and increasing the content of stilbenes, such as MYB, ERF, NAC, and bZIP (Fig. S2). Co-regulation also exists between different TFs (Vannozzi et al. 2018). In *V. davidii*, VdMYB1 activates *VdSTS2* and stimulates the response to pathogen infection (Yu et al. 2019). In *V*. *quinquangularis*, VqMYB15 interacts with VqNAC44, and their co-overexpression activates *VqSTS* (VIT_16s0100g01200) and increases the production of stilbene compounds (Wang et al. 2024). VqMYB35 activates the expression levels of *VqSTS15*, *VqSTS28*, *VqSTS42*, and *VqSTS46*, and co-overexpression of VqERF114 and VqMYB35 results in elevated *VqSTS* expression and increased stilbene synthesis (Wang and Wang 2019b). VqMYB154 activates *VqSTS9*, *VqSTS32*, and *VqSTS42* by directly binding to their promoters and enhancing grapevine resistance to powdery mildew (Jiang et al. 2021). The expression of VvMYB9/14/15a/107 also increases the production of stilbene compounds (Kiselev et al. 2017). Interestingly, resveratrol inhibits the over-accumulation of resveratrol to balance metabolic costs through VvMYB14-VvSTS15/21-Res-VvWRKY8 and VvMYB14–VvWRKY8–VvMYB30 regulatory loops under UV stress in grape (Jiang et al. 2019; Mu et al. 2023b). However, whether these regulatory loops also play a role in responding to fungal diseases remains unclear.

For other TFs, Alfin-like transcription factor VqAL4 also activates *VqNSTS4* expression and increases grape resistance to powdery mildew (Yan et al. 2023). In *V*. *quinquangularis*, except for VqWRKY56, VqbZIPC22 can bind to the VvANR promoter, promote proanthocyanidin accumulation, and improve grape resistance to powdery mildew (Wang et al. 2023d). In *V. amurensis*, VaERF16 interacts with VaMYB306, and overexpression of *VaERF16* or *VaMYB306* in grape leaves increases resistance to *B*. *cinerea*. VaERF16 can bind to the *VaPDF1.2* promoter but not VaMYB306, but the VaERF16–VaMYB306 complex promotes elevated transcript levels of *VaPDF1.2* (Zhu et al. 2022b). In *V. vinifera*, VvNAC72 negatively modulates methylglyoxal-associated ROS by repressing transcript levels of *VvGLYI-4* and then enhances grape resistance to downy mildew (Li et al. 2021a).

### Resistance-related proteins

The first resistance genes, *MrRNU1* and *MrRPV1*, were cloned from wild grapevine species *M*. *rotundifolia* and transferred to susceptible *V*. *vinifera* cultivars, and they confer resistance to major pathogens of powdery mildew and downy mildew (Feechan et al. 2013). Subsequent transcriptome analysis showed that WRKY and MYB TFs strongly co-express with *STS* genes, and many resistance-related genes are upregulated in *MrRPV1*-transgenic grapes against *P*. *viticola* (Qu et al. 2021). These results indicate that *STS*s and other resistance-related genes are downstream of NLR activation. Functional analysis found that overexpression of *VqNSTS3*, *VqSTS6*, and *VpSTS29/STS2* in *V*. *vinifera* increases resistance to powdery mildew (Liu et al.^2019^; ^2023^; Xu et al. 2019). Moreover, overexpression of *VpPR4-1* and *VpPR10*.*1* from wild Chinese grape *V*. *pseudoreticulata* in *V*. *vinifera* enhances powdery mildew resistance (Dai et al. 2016; Ma et al. 2018), whereas knockout *VvPR4b* in *V*. *vinifera* increases susceptibility to downy mildew (Li et al. 2020a). The ectopic expression of broad-spectrum resistance genes also improves grape resistance. For example, overexpression of the *Arabidopsis* resistance gene *RPW8.2* and rice chitinase gene *RCC2* enhances the resistance of transgenic grapevines to powdery mildew (Hu et al. 2018; Yamamoto et al. 2000).

Ubiquitination is a key post-translational modification during plant–pathogen interactions (Ma et al. 2021b; Sharma et al. 2023). In wild *V*. *pseudoreticulata*, three ubiquitin ligase genes, *VpRH2*, *VpEIRP1*, and *VpPUB23*, are associated with significant resistance to *E. necator* via overexpression (Wang et al. 2017; Yu et al. 2013; Zhou et al. 2014). Specifically, VpRH2 interacts with a glycine-rich RNA-binding protein VpGRP2A but does not promote the degradation of VpGRP2A, which enhances the expression of VpRH2 (Wang et al. 2017). *VpEIRP1* contributes to resistance by mediating the degradation of the negative regulator VpWRKY11 (Yu et al. 2013). In *V*. *vinifera*, Recent findings elucidated that *VvPUB26* ubiquitinates *VvWRKY24*, which inhibits PA biosynthesis. Overexpression of *VvPUB26* in transgenic grapevines increases PA content and resistance to powdery mildew (Zhao et al. 2024). These results indicate that ubiquitination plays a crucial role in grapevine disease resistance as a post-translational protein modification.

## Innovative approaches to improve genetic transformation and gene editing efficiency in grapevine

*Agrobacterium*-mediated transformation in grapevine is widely used via somatic embryogenesis (SE) and organogenesis, and stable genetic transformation in grapevine has been summarized by Zhang et al. (2021) since 2001. Recent studies proposed a combination of SE and organogenesis. Capriotti et al. (2023) developed an efficient protocol based on cotyledons and hypocotyls from somatic embryos, and the transformation efficiency of cotyledons and hypocotyls is 14% and 12% on high-regeneration culture medium, respectively. Another approach is to obtain transgenic grapevine plants via transfection and subsequent regeneration of protoplasts isolated from embryogenic callus (Najafi et al. 2023; Tricoli and Debernardi 2023). Specifically, regeneration based on individual cells can greatly avoid the formation of chimeric plants during the genetic transformation of grapevine (Scintilla et al. 2022). Zhang et al. (2023a) established a stable genetic transformation system using grape immature zygotic embryos from ‘Chardonnay’ seeds, and the highest transformation efficiency is 12.38%. Similar to other plants, low efficiency and genotype dependence are problems faced in the genetic transformation of grapes. Therefore, taking advantage of development regulators or morphogenic TFs such as *Wuschel2* (WUS2), *Baby boom* (BBM), *Growth-regulating factor4* (GRF4), and its cofactor *GRF-interacting factor1* (GIF1) will improve regeneration and transformation in grape (Lee and Wang 2023). Although there is no data support on grapes to date, epicotyls transformed with the grape *GRF-GIF* chimera were reported to display a 4.7-fold increase in citrus regeneration frequency compared with the empty vector (Debernardi et al. 2020).

Since 2016, CRISPR/Cas9-mediated gene editing in grape has been reported in different explants, such as embryogenic callus and cells, with editing efficiency ranging from 0.1% to 100% (Ren et al. 2022). Wang et al. (2018) obtained 22 *VvWRKY52* gene-edited grape plants from 72 T-DNA-inserted plants using the CRISPR/Cas9 system with 31% editing efficiency. Subsequent genome sequencing further confirmed that only one off-target indel mutation was identified from 3272 potential off-target sites (Wang et al. 2021), suggesting that the CRISPR/Cas9 system has high specificity in grapevine. Although CRISPR-mediated gene editing has been applied successfully in grape, there is still room for optimization to improve editing efficiency. Ren et al. (2019) designed different GC contents of single-guide RNA (sgRNA) to optimize CRISPR/Cas9 editing efficiency, and the results found that 65% GC content of sgRNA yields the highest editing efficiency. Subsequently, higher mutation rates and biallelic rate of *VvPDS* were observed in transgenic grapevine plants using the grape promoters VvU3 and VvU6 than using the AtU6 promoter via optimizing the CRISPR/Cas9 system (Ren et al. 2021). These results indicated that grape-specific promoters are a suitable choice for improving gene editing efficiency in grapes. Gene encoding *tonoplastic monosaccharide transporter1* (*TMT1*) was effectively knocked out in embryogenic cells of ‘41B’ using the CRISPR/LbCas12a-mediated gene editing system, and the editing efficiencies of *TMT1* increased from 29.9% to 44.6% after heat treatment (Ren et al. 2023). Recently, Yang et al. (2024) optimized prime editor and successfully obtained *VvDXS1*-edited grapevine lines with an editing efficiency of more than 50% in the table grape *V. vinifera* cv. Scarlet Royal.

DNA-free genome editing using CRISPR/Cas9 ribonucleoprotein (RNP) complexes was applied in grape protoplasts to eliminate the risk of integrating gene editing vectors into the genome (Malnoy et al. 2016; Osakabe et al. 2018). The regeneration efficiency of transfected protoplasts is 23% for Cas9-sgRNA RNP2 and 34% for Cas9-sgRNA RNP4 (Najafi et al. 2023). A recent report showed that lipofectamine-mediated transfections are used to deliver RNPs in grape protoplast and obtain edited plants of Nebbiolo grapevines (Gambino et al. 2024). Like other crops, genetic transformation is highly dependent on genotypes in grapevine. However, Tricoli and Debernardi (2023) developed an efficient protoplast-based genome editing method for Thompson Seedless, Colombard, and Merlot varieties, as well as the wild relative *V*. *arizonica* with RNP complexes. In addition to DNA editing, RNA editing has been introduced into grapevine using the CRISPR/FnCas9 and CRISPR/LshCas13a systems through transient delivery (Chen et al. 2021; Jiao et al. 2022), providing a new method for obtaining stable gene-edited grape plants. Thus, new avenues of effectively generating edited or DNA-free edited grapevine lines are powerful tools for molecular breeding of grape disease resistance.

## Strategies to improve grape resistance to fungal diseases

Resistant quantitative trait locus (QTL) development in grapes is an important pathway for developing disease-resistant molecular markers and screening candidate disease-resistant genes. In the past few decades, many fungal disease-resistant loci have been identified, such as resistance locus *Rgb1* for black rot (Rex et al. 2014), loci *Rda1* and *Rda2* for *Phomopsis* cane spot (Barba et al. 2018), *Cgr1* for ripe rot (Fu et al. 2019), two loci for white rot (Su et al. 2021; Li et al. 2023), 37 *Rpv* loci for downy mildew, and 16 *Ren* and *Run* loci for powdery mildew (Pirrello et al. 2023; Sosa-Zuniga et al. 2022) in different genetic backgrounds, especially from wild accessions and species. Most *R* loci regions in grapes contain putative disease resistance (*R*) genes with nucleotide-binding sites (NBSs) and leucine-rich repeat (LRR) domains. Interestingly, *R* genes are clustered in tandem repeats in grape genomic regions (Feechan et al. 2013; Sosa-Zuniga et al. 2022). Abundant *R* loci have been found in wild grapes, which are important genetic resources of genetic diversity and disease resistance for cross breeding (Foria et al. 2022; Margaryan et al. 2023; Qiu et al. 2015). However, the unexpected traits and tightly linked loci with unfavorable traits in wild grapes require multiple generations of backcrossing to remove or break them. This can result in a long breeding cycle and time-consuming process. For grape cultivars, at least four *R* loci for resistance to downy mildew and powdery mildew are carried by Bronner, Cabernet cortis, Calardis blanc, Regent, Seyval blanc, and Solaris (Pirrello et al. 2023; Salotti et al. 2022). Therefore, crossing between cultivars with *R* loci is a feasible method for aggregating multiple resistances to fungal diseases. From the perspective of application, marker-assisted selection (MAS) and genomic selection are beneficial to the selection of parents and the screening of disease-resistant offspring (Katula-Debreceni et al. 2010; Brault et al. 2022). It is also necessary to conduct research on gene function from a research perspective before application, such as transgenesis and gene editing. How to screen candidate genes becomes particularly important.

**Fig. 4:**
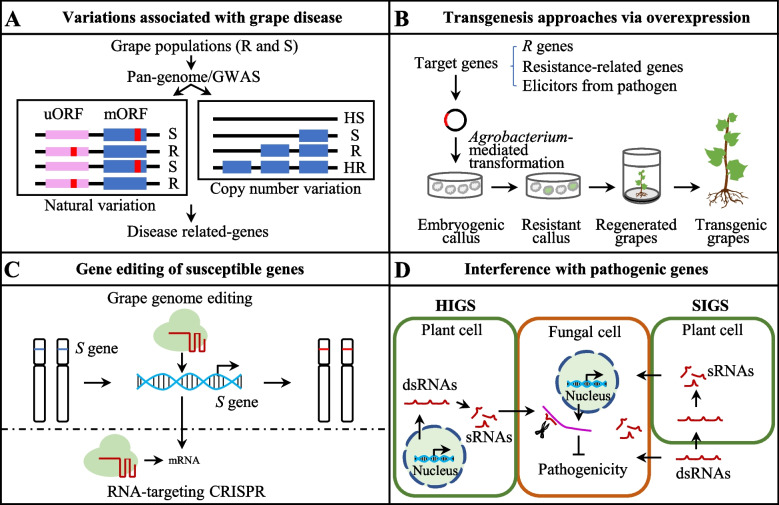
Strategies to enhance grape resistance to pathogenic fungi and oomycetes. A, Identification of structural variant (SV) and copy number variation (CNV) associated with grape resistance. B, Overexpression of R genes or resistance-related genes based on grape genetic transformation. C, CRISPR-mediated DNA or RNA editing of grape-susceptible genes. D, RNA silencing of virulence genes of grape pathogenic fungi by host-induced gene silencing (HIGS) or spray-induced gene silencing (SIGS)

Natural variation is widely present in different plant species due to long-term natural and artificial selection, such as structural variations (SVs) and copy number variations (CNVs), which may be involved in disease resistance (Dolatabadian et al. 2017; Wang et al. 2023a) (Fig. 4A). New genomic approaches have facilitated rapid identification of the candidate resistance gene by pan-genomics and genome-wide association studies (GWAS) in plants (Dracatos et al. 2023). For example, pan-genome analysis of 13 *Malus* accessions revealed an SV gene *MdMYC2-like* associated with disease resistance (Wang et al. 2023c). In grapevine, GWAS analysis identified 44 SNP loci that are responsible to resistances to phylloxera, root-knot nematodes, and abiotic stresses (Wang et al. 2023b). Pan-genome analysis found genomic variations associated with resistances to Pierce’s disease (Cochetel et al. 2023), but little information is reported for fungal diseases. The candidate genes obtained through this approach require subsequent functional validation and further identification in different genotypes.

Compared with conventional breeding, transgenesis opens a new toolkit for grape resistance fungal disease breeding in cost-effective ways: (i) overexpression of *R* gene, resistance-related genes, or elicitors of pathogen, (ii) gene editing or interfering with the grape susceptible (*S*) gene, and (iii) silencing fungal effectors or virulence genes (Fig. 4B-D). Two *R* genes, *MrRUN1* and *MrRPV1*, have been found to confer broad-spectrum resistance to most powdery and downy mildew isolates, but not including the Musc4 isolate from Georgia (Feechan et al. 2013). Although the broad-spectrum resistance of grapes is generally achieved through the cumulative effect of multiple resistance genes, further exploration is needed on the interaction mechanism of *R*-*Avr* pairs. In addition to the *R* gene, downstream genes including calcium ion signaling, TFs, and coding genes of pathogenesis-related protein are involved in grape fungal disease resistance (Table 2 and S3). Some effectors from pathogenic fungi do not only interfere with the immune response of grapes but also act as elicitors. The *P. viticola* effector PvAVH53 interacts with grapevine nuclear import factor importin alphas (VvImpα and VvImpα4) in the nucleus and triggers cell death (Chen et al. 2021). Increased accumulation of salicylic acid and ROS is observed in grapevine after application of exogenous purified *P. viticola* effector PvAvh77, suggesting that it has the potential to serve as an inducer of grape immunity (Fu et al. 2023a). Another *P. viticola* effector PvCRN11 enhances grapevine resistance to downy mildew via overexpression in grape lines resulting from the accumulation of salicylic acid and ROS (Fu et al. 2023b). The prerequisite for transgenesis in grape is to obtain resistance genes using efficient and precise approaches, especially to achieve broad-spectrum disease resistance.

Editing or silencing of susceptible (*S*) genes using CRISPR/Cas9 or RNAi is also an important way to improve grape disease resistance (Pirrello et al. 2023). *Mildew Locus O* (*MLO*) genes are typical class *S* genes to powdery mildew. In grapevine, *VvMLO3*-edited grapevine lines enhance resistance to powdery mildew (Wan et al. 2020). Similarly, RNAi lines of *VvMLO6*/*7*/*11* reduce powdery mildew severity up to 77% (Pessina et al. 2016). For grape downy mildew, *VviDMR6-1/2*, *VvWKRY40*, *VpPsbP*, and *VpBPA1* are susceptibility factors in grape (Giacomelli et al. 2023; Liu et al. 2021; Ma et al. 2021a; Yin et al. 2022). *Downy Mildew Resistant 6* (*DMR6*) is known as an *S* gene for downy mildew in many crops. In grapevine, double mutant *dmr6-1*_*2* significantly reduces susceptibility to downy mildew due to the accumulation of endogenous SA when compared with the wild type, whereas single mutations of either *VviDMR6-1* or *VviDMR6-2* do not display any reduction (Giacomelli et al. 2023). For grape gray mold, *VvWRKY52*-edited grapevine lines enhance resistance to *B*. *cinerea*, especially in biallelic mutant lines (Wang et al. 2018). CRISPR/Cas9 can be used to construct a genome-wide mutant library in grape to help find additional *S* genes. Multi-*S* gene knockout is an effective way to obtain resistant grapes to various fungal diseases.

Another strategy for improving grape disease resistance is to silence fungal virulence genes such as effectors using host-induced gene silencing (HIGS) or spray-induced gene silencing (SIGS) (Lopez-Gomollon and Baulcombe 2022; Wang and Jin 2017). The growth of powdery mildew mycelium in grapevine leaves is inhibited after silencing the *E. necator* effector CSEP080. In addition, silencing virulence *PvDCL1* and *PvDCL2* of *P*. *viticola* reduces the disease progress rate using SIGS (Haile et al. 2021). A similar approach was used for powdery mildew management by targeting six *E. necator* genes, leading to the reduction of spore production on grapevine (McRae et al. 2023). One important consideration for this strategy is how to avoid off-targets and to evaluate the efficiency and cost.

## Conclusions

Recent advances have been summarized in our understanding of the molecular basis of grape–fungal disease interactions and disease resistance in grapevine. Currently, extensive research focused on grape powdery mildew and downy mildew. Given the diversity of grape fungal diseases, further exploration of interaction mechanisms, QTL detection, and mining of disease-resistant genes related to other grape fungal diseases is needed. In particular, the recognition of grape receptors (PRRs) and MAMPs of pathogenic fungi, as well as interactions of *R*–*Avr*, remains largely unknown. The exploration of *R* genes or resistance-related genes from wild resistant grapes and gene editing *S* genes in susceptible grapes are important for enhancing the disease resistance of susceptible cultivars. However, one important consideration for transgenesis is how to improve the efficiency of genetic transformation and gene editing in grape. With the improvement of various systems and the research innovations in the future, these questions in grapevine will be solved. For example, a pooled effector library may be used to rapidly identify *Avr* genes, whereas a pooled CRISPR library may help rapidly identify disease resistance genes.

## Supplementary Information

Supplementary Material 1: Table S1. List of pathogenic fungi in grapevine. Table S2. Sequenced genome list of pathogenic fungi in grapevine. Table S3. List of grape transcription factors associated with resistance and susceptibility to pathogens

Supplementary Material 2: Fig. S1. Functions of grape transcription factors (TFs) in biotic stresses. The function of TFs was identified in grapevines or other plants. Ended arrows represent resistance, and arrows represent susceptibility. Fig. S2. Model for grape regulating *STSs* and accumulation of resveratrol under biotic stress and UV stress. Grape *STS* genes were regulated by WRKY, MYB, ERF, bZIP, and Alfin-like TFs (pink box). Expression of TFs and co-expression of TF complexes can increase the content of resveratrol (blue box). VvMYB30 regulating *VvSTSs* through VvMYB14 and VvWRKY8 regulatory loop to control UV-induced stilbene biosynthesis in grapevine (green box). Arrows represent promotion. Ended arrows represent suppression.

## Data Availability

Not applicable.

## Abbreviations

CNVs: Copy number variations
CRISPR: Clustered regularly interspaced short palindromic repeats
DAMPs: Damage-associated molecular patterns
ET: Ethylene
ETI: Effector-triggered immunity
GTDs: Grapevine trunk diseases
GWAS: Genome-wide association studies
HIGS: Host-induced gene silencing
HR: Hypersensitive response
JA: Jasmonic acid
MAPKs: Mitogen-activated protein kinases
MAS: Marker-assisted selection
MeJA: Methyl jasmonate
NLRs: Nucleotide-binding leucine-rich repeat proteins
PAMPs: Pathogen-associated molecular patterns
PCD: Programmed cell death
PCWDEs: Plant cell wall-degrading enzymes
PRRs: Pattern recognition receptors
PTI: Pattern − triggered immunity
QTL: Quantitative trait locus
RNP: Ribonucleoprotein
ROS: Reactive oxygen species
SA: Salicylic acid
SIGS: Spray-induced gene silencing
STSs: Stilbene synthases
SVs: Structural variations
TFs: Transcription factors
