# Supplementary material for: Advances in the molecular mechanism of grapevine resistance to fungal diseases
Source: Mol Hortic. 2025 Jan 2;5:1. doi: 10.1186/s43897-024-00119-x (PMC11694456; doi:10.1186/s43897-024-00119-x)
Supplement: Supplementary file 2 — Supplementary Material 2: Fig. S1. Functions of grape transcription factors (TFs) in biotic stresses. The function of TFs was identified in grapevines or other plants. Ended arrows represent resistance, and arrows represent susceptibility. Fig. S2. Model for grape regulating STSs and accumulation of resveratrol under biotic stress and UV stress. Grape STS genes were regulated by WRKY, MYB, ERF, bZIP, and Alfin-like TFs (pink box). Expression of TFs and co-expression of TF complexes can increase the content of resveratrol (blue box). VvMYB30 regulating VvSTSs through VvMYB14 and VvWRKY8 regulatory loop to control UV-induced stilbene biosynthesis in grapevine (green box). Arrows represent promotion. Ended arrows represent suppression. [file 43897_2024_119_MOESM2_ESM.pdf]

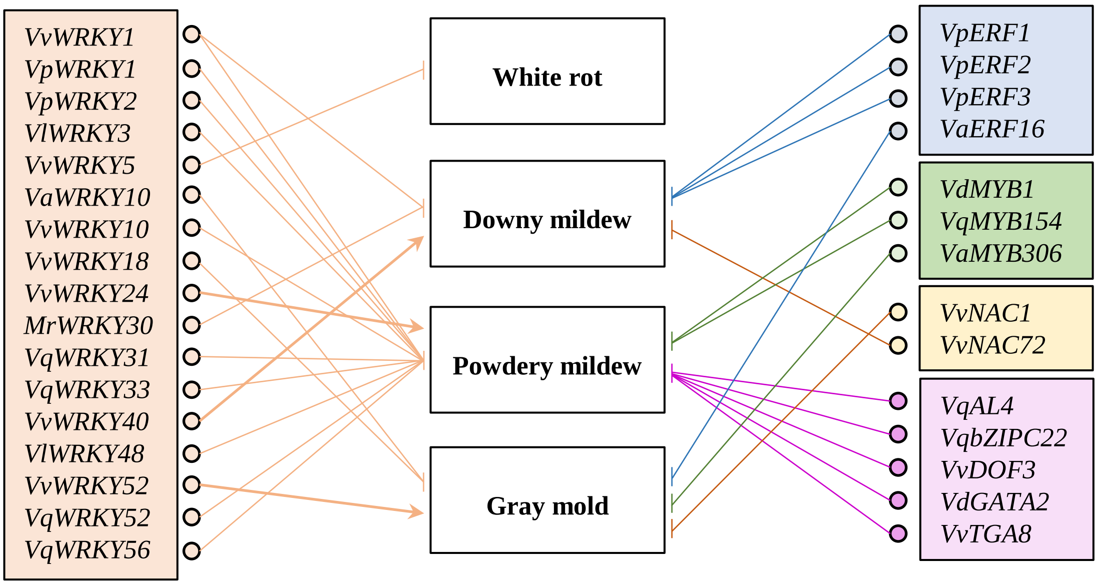

Fig. S1 Functions of grape transcription factors (TFs) in biotic stresses. The function of TFs was identified in grapevines or other plants. Ended arrows represent enhanced resistance, and arrows represent weakened resistance.

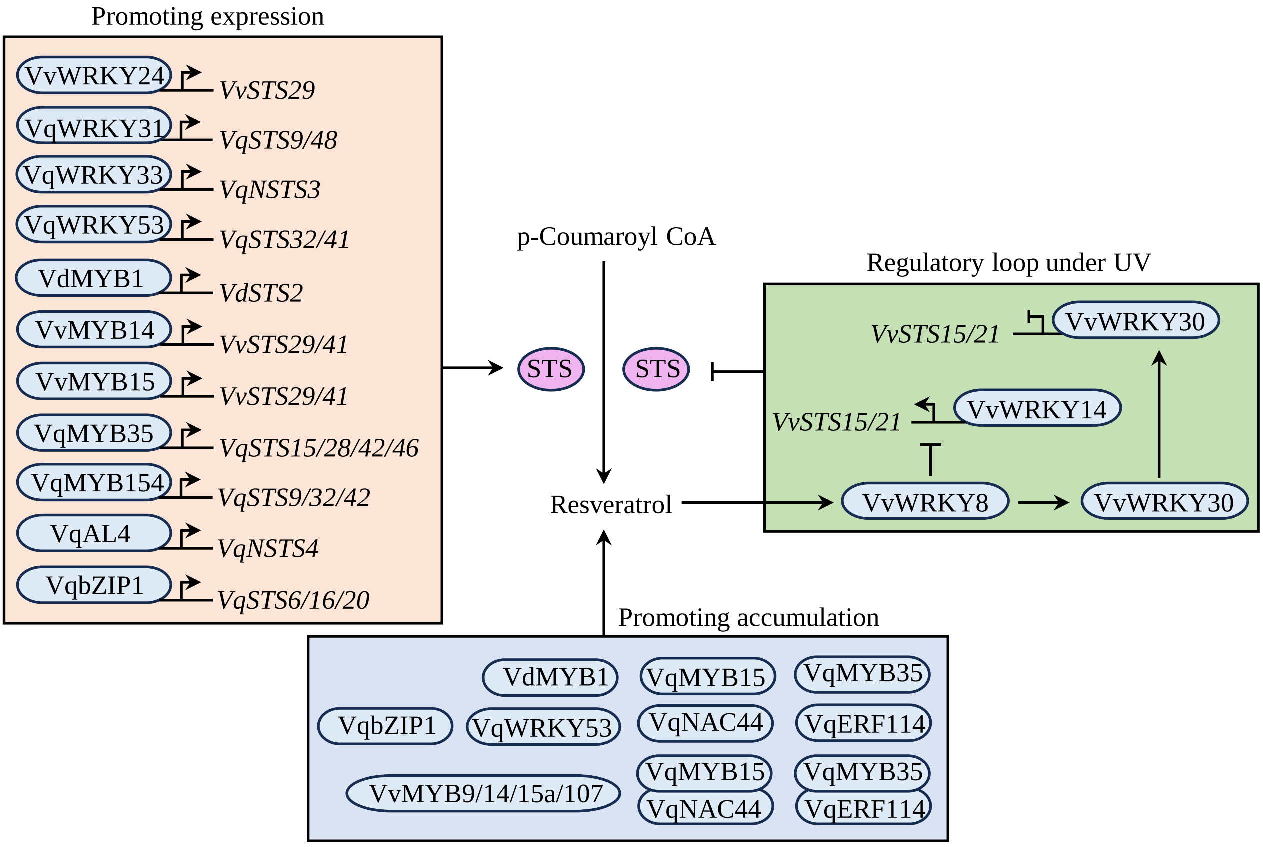

Fig. S1 Model for grape regulating *STSs* and accumulation of resveratrol under biotic stress and UV stress. Grape *STS* genes were regulated by WRKY, MYB, ERF, bZIP, and Alfin-like TFs (pink box). Expression of TFs and co-expression of TF complexes can increase the content of resveratrol (blue box). VvMYB30 regulating *VvSTSs* through VvMYB14 and VvWRKY8 regulatory loop to control UV-induced stilbene biosynthesis in grapevine (green box). Arrows represent promotion. Ended arrows represent suppression.
